# Occult Urinary Incontinence Treatment: Systematic Review and Meta-analysis—Brazilian Guidelines*

**DOI:** 10.1055/s-0038-1676842

**Published:** 2019-02

**Authors:** Priscila Katsumi Matsuoka, Rodrigo Aquino Castro, Edmund Chada Baracat, Jorge Milhem Haddad

**Affiliations:** 1 Faculty of Medicine, Universidade de São Paulo, São Paulo, SP, Brazil; 2 Paulista Medical School, Universidade Federal de São Paulo, São Paulo, SP, Brazil

**Keywords:** occult urinary incontinence, systematic review, pelvic organ prolapse, meta-analysis, stress urinary incontinence, incontinência urinária oculta, revisão sistemática, prolapso de órgãos pélvicos, metanálise, incontinência urinária de esforço

## Abstract

**Objective** To evaluate if performing anti-incontinence procedures during surgical anterior and/or apical prolapse correction in women with asymptomatic urinary incontinence (UI) may prevent stress urinary incontinence (SUI) postoperatively.

**Methods** We have performed a systematic review of articles published in the PubMed, Cochrane Library, and Lilacs databases until March 31, 2016. Two reviewers performed the data collection and analysis, independently. All of the selected studies were methodologically analyzed. The results are presented as relative risk (RR), with a 95% confidence interval (CI).

**Results** After performing the selection of the studies, only nine trials fulfilled the necessary prerequisites. In the present review, 1,146 patients were included. Altogether, the review included trials of three different types of anti-incontinence procedures. We found that performing any anti-incontinence procedure at the same time of prolapse repair reduced the incidence of SUI postoperatively (RR = 0.50; 95% CI: 0.28–0.91). However, when we performed the analysis separately by the type of anti-incontinence procedure, we found different results. In the subgroup analysis with midurethral slings, it is beneficial to perform it to reduce the incidence of SUI (RR = 0.08; 95% CI: 0.02–0.28). On the other hand, in the subgroup analysis with Burch colposuspension, there was no significant difference with the control group (RR = 1.47; 95% CI: 0.28–7.79]).

**Conclusion** Performing any prophylactic anti-incontinence procedure at the same time as prolapse repair reduced the incidence of SUI postoperatively. The Burch colposuspension did not show any decrease in the incidence of SUI postoperatively.

## Abstract (pt)

**Resumo:**

**Objetivo:** Avaliar se a realização de procedimentos anti-incontinência durante a correção cirúrgica do prolapso anterior e/ou apical em mulheres assintomáticas para incontinência urinária (IU) pode prevenir a incontinência urinária de esforço (IUE) no pós-operatório.

**Métodos:** Foi realizada uma revisão sistemática dos artigos publicados nas bases de dados PubMed, Cochrane Library e Lilacs até o dia 31 de março de 2016. Dois revisores realizaram a coleta e a análise de dados, de forma independente. Todos os estudos selecionados foram analisados metodologicamente. Os resultados estão apresentados como risco relativo (RR), com 95% de intervalo de confiança (IC).

**Resultados:** Após a seleção do estudo, apenas nove estudos preencheram os pré-requisitos necessários. Nesta revisão, 1.146 pacientes foram incluídos. No total, a revisão incluiu ensaios de três tipos diferentes de procedimentos anti-incontinência. Descobrimos que realizar qualquer procedimento anti-incontinência ao mesmo tempo que o reparo do prolapso reduziu a incidência de IU no pós-operatório (RR = 0,50; IC 95%: 0,28–0,91). No entanto, quando fizemos a análise separadamente pelo tipo de procedimento anti-incontinência, encontramos resultados diferentes. Na análise de subgrupos com slings miduretrais, é benéfico realizá-lo para reduzir a incidência de IU (RR = 0,08; IC 95%: 0,02–0,28). Por outro lado, na análise de subgrupo com colposuspensão de Burch, não houve diferença significativa com o grupo controle (RR = 1,47; IC 95%: 0,28–7,79]).

**Conclusão:** A realização de qualquer procedimento profilático anti-incontinência ao mesmo tempo que o reparo do prolapso reduziu a incidência de IUE no pós-operatório. A colposuspensão de Burch não mostrou diminuição na incidência de IUE no pós-operatório.

## Introduction

In Brazil, in the last 50 years, there was an increase of 27 years in life expectancy, according to the Brazilian Institute of Statistics and Geography (IBGE, in the Portuguese acronym), Consequently to the continuous aging process of the population, we have had a great change in the most prevalent diseases in the Brazilian population, in which chronic diseases became more frequent than contagious diseases. In this scenario, it is estimated that 35% of the climacteric women in a Southeastern city, Campinas, in the state of São Paulo, present urinary incontinence (UI).^1^ ^2^ ^3^ ^4^ ^5^ ^6^ ^7^

According to the International Continence Society (ICS) and to the International Urogynecological Association (IUGA), occult or latent UI is defined as stress urinary incontinence (SUI) only when observed after the reduction of a coexistent prolapse.^1^ It is when a clinically continent patient with severe prolapse develops UI after the reduction of the genital prolapse or after the surgical correction of the prolapse.^1^ This is believed to be attributable to urethral kinking or even to extrinsic compression of the urethra by the genital prolapse.^2^

Therefore, when the obstructive factor is removed, some patients may present loss of urine. Some authors recommend treating latent UI in a second procedure, after correcting the genital prolapse.^3^ ^4^ Nevertheless, with the purpose of reducing the costs involved in a new hospitalization and the risks arising from a new surgical procedure, some authors have conducted a considerable series of studies assessing the effectiveness of correcting occult SUI and treating genital prolapse in a single procedure.^3^ ^4^

Despite these facts, there is no consensus in the literature about the preventive treatment for UI at the same time as prolapse surgical repair in asymptomatic patients.^5^ Some authors recommend that the treatment of occult UI should be performed in a second surgery, after the prolapse surgical treatment, considering that the anti-incontinence procedures are not without any complications, such as urinary retention, *de novo* overactive bladder, bladder perforation, and bleeding. However, the most relevant point of this view is that no an anti-incontinence procedure will be performed without necessity.^5^ In addition, the study conducted by Costantini et al^6^ showed that there was no benefit in performing Burch colposuspension associated with sacrocolpopexy to prevent UI after 1 year. And due to the potential increase in the frequency of patients with pelvic organ prolapse and occult UI who will require treatment, the Brazilian Society of Obstetrics and Gynecology sees the urgency to move forward and establish clinical guidelines for Brazilian gynecologists who care for these women. The purpose of the present study, by recommending evidence-based data, is to assist healthcare professionals in the management, in the diagnosis, in the clinical assessment, and in the optimization of the efficacy of the treatment of occult urinary incontinence.

The evaluation of the need to perform anti-incontinence procedures during surgical anterior and/or apical prolapse correction in women with asymptomatic UI may prevent SUI postoperatively.

## Methods

The present systematic review of occult UI is part of a task force group to design the Brazilian guidelines in Urogynecology for The Brazilian Society of Obstetrics and Gynecology (FEBRASGO, in the Portuguese acronym). The present review is endorsed by the Urogynecology and Vaginal Surgery Committee of the FEBRASGO.

We have performed a systematic review of the literature and, when possible, we have also performed a meta-analysis of the topic. The systematic review was performed only with randomized controlled clinical trials. We have selected studies with clinically continent adult women with anterior genital prolapse or vaginal vault prolapse classified as grade 3 or higher, according to the Pelvic Organ Prolapse Quantification (POP- Q) system, who underwent surgical correction of genital prolapse. The patients were submitted to prolapse repair only and were compared with patients with the correction associated with an anti-incontinence procedure. The primary outcome evaluated was the incidence of SUI after the surgical correction of the prolapse or the need of surgical treatment for SUI. The secondary outcome comprised adverse events related to these surgical procedures.

### Strategy for the Literature Review

We have performed a systematic literature review of articles published in the PubMed, Cochrane Library, and Lilacs databases until March 31, 2016. For this purpose, we developed a research strategy based on descriptors and synonyms for UI and genital organ prolapse.

Two independent researchers performed the study screening process. At the end of this stage, their selections were compared, and discrepant cases were solved by consensus. In cases of discordance, a third researcher conducted a discussion and solved the discrepancies. All of the selected studies were assessed for their methodological quality and risk of bias by two researchers, using the technique developed by Jadad et al^8^ in 1996 and the levels of evidence established by the Oxford Centre for Evidence-Based Medicine.^8^ ^9^ The evaluation of the methodological quality was used to analyze as a predictor of the strength of the evidence provided by each study rather than as an inclusion criterion for the literature review.

## Statistical Analysis

The non-continuous variables were expressed as relative risk (RR), and the continuous variables as weighted mean difference, both with a confidence interval (CI) of 95%. The value used for rejecting the null hypothesis was 5%. After the systematic review, a meta-analysis was performed using the RevMan software, Version 5.1 (The Nordic Cochrane Center, The Cochrane Collaboration, Copenhagen, Denmark). Dichotomous variables were analyzed using the Mantel–Haenszel statistical method in a random-effects model of RR with 95% CI. Study heterogeneity was calculated by I^2^. According to Higgins et al,^10^ consider of low, moderate, and high to I^2^ values of 25%, 50%, and 75% Articles were divided into subgroups according to the type of anti-incontinence procedure performed.

## Results

**Fig. 1:**
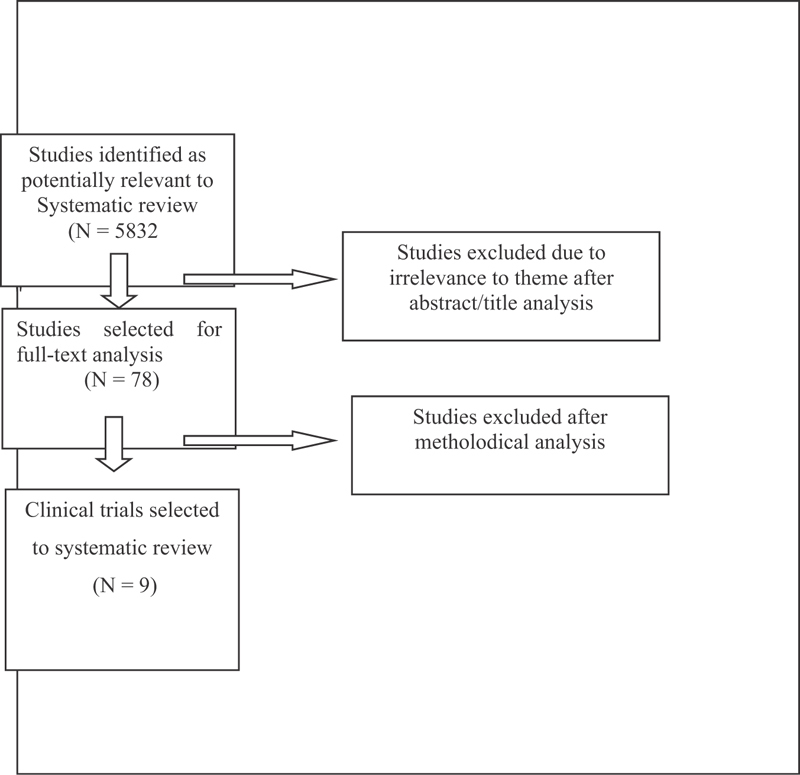
Preferred reporting items for systematic reviews and meta-analyses (PRISMA) fluxogram

After the primary selection, we selected 78 articles for full-text analysis, but only 9 eligible trials fulfilled the necessary prerequisites for the present systematic review (Fig. 1).

In the present systematic review, 1,146 patients were included. Altogether, the review included trials of four different types of anti-incontinence procedures: midurethral sling (retropubic and transobsturator), Burch colposuspension, needle colposuspension, and cystopexy with posterior pubourethral ligaments plication.^10^ ^11^ ^12^ ^13^ ^14^ ^15^ ^16^ ^17^ ^18^ ^19^ Kelly plications were not considered an anti-incontinence procedure.

In the analyzed studies, the surgeries for prolapse correction were not standardized and included the following techniques: vaginal hysterectomy, anterior repair, paravaginal anterior repair, posterior repair, sacrospinous ligament vaginal suspension, bilateral iliococcygeus vaginal suspension, culdoplasty, sacroxolpopexy, perineoplasty, colpocleisis, and prolapse repair surgery with Prolift (Ethicon by Johnson & Johnson) meshes (Table 1).

**Table 1:** We found only two studies classified with the highest score according to the Jadad scale. However, five studies were well-designed randomized clinical trials according to the Oxford levels of evidence

	Author	Year	Oxford levels of evidence	Jadad Score	Experimental (*n*)	Control (*n*)	Follow-up	Outcome	Loss to follow-up
1	Wei et al.^11^	2012	1b	5	POP + TVT (165)	POP (172)	3 mo and 1y	SUI	Experimental: 3/165 Control: 7/172 (1y)
2	Khelaia and Khelaia^12^	2008	2b	1	AC + TVT (46)	AC (46)	52 mo – average	SUI	Not informed
3	Schierlitz et al.^13^	2014	1b	1	POP + TVT (37)	POP (43)	6 mo and 27 mo	Surgery for SUI	Experimental: 6/37 Control: 7/43
4	Fuentes^14^	2011	2b	1	POP + TVTo (27)	POP (33)	6 mo and 15 mo	Surgery for SUI	Not informed
5	Busacchi and Paganotto^15^	2012	2b	2	AC + TOT (46)	AC (53)	1 mo, 6 mo, 1y, and 2y	SUI	Not informed
6	Brubaker et al.^18^	2008	1b	5	SC + Burch (157)	SC (165)	3 mo, 1y, 2y, and 7y	SUI	Experimental: 5/157 Control: 12/165 (1y)
7	Costantini et al.^6^	2007	2b	1	SC + Burch (34)	SC (32)	3 mo, 6 mo, and 1y	SUI	No loss (6 mo)
8	Colombo et al.^19^	1996	1b	3	CP + PPL (50)	CP (52)	1y	SUI	Not informed
9	van der Ploeg et al.^20^	2016	1b	4	POP + MUS (48)	POP (43)	1y	SUI	Experimental: 0/43 Control: 1/48 (1y)

Abbreviations: AC, anterior colporrhaphy; CP, cystopexy; mo, month; POP, pelvic organ prolapse; PPL, posterior pubourethral ligaments; SC, sacrocolpopexy; SUI, stress urinary incontinence; TOT, out-in transobturator sling; TVT, retropubic sling; TVTo, in-out transobturator sling; y, year.

**Fig. 2:**
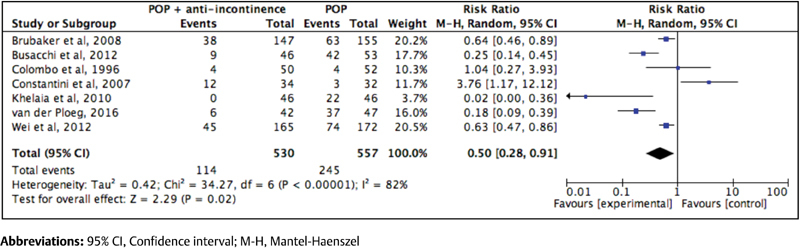
Forest plot of the global effect of anti-incontinence procedures on the incidence of urinary incontinence after surgical treatment of pelvic organ prolapse

A meta-analysis was performed only with variables common to studies and the same scale of quantification. First, we analyzed the global effect of anti-incontinence procedures in terms of incidence of UI after the surgical procedure (Fig. 2) It is important to highlight that we have only included seven clinical trials in this first analysis, because they had the same outcome.^6^ ^11^ ^12^ ^15^ ^16^ ^17^ ^18^ ^19^ ^20^

**Fig. 3:**
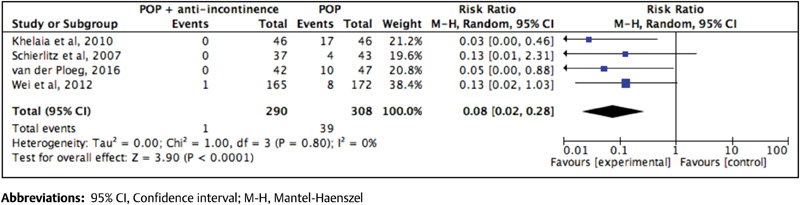
Forest plot of the effect of mid-urethral slings on the incidence of urinary incontinence after surgical treatment of pelvic organ prolapse

**Fig. 4:**
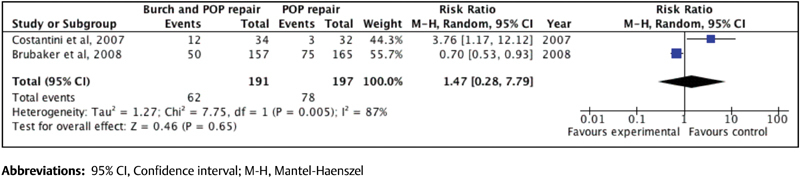
Forest plot of the effect of Burch colposuspension on the incidence of urinary incontinence after surgical treatment of pelvic organ prolapse

We found that performing any prophylactic anti-incontinence procedure at the same time as the prolapse repair reduced the incidence of SUI postoperatively (RR = 0.50; 95% CI: 0.28–0.91).^6^ ^11^ ^12^ ^15^ ^16^ ^17^ ^18^ ^19^ ^20^ However, when the types of anti-incontinence procedure were analyzed separately, we found different results. If we evaluate separately the anti-incontinence procedures performed concomitantly with prolapse repair, the results differ considerably (RR = 0.08; 95% CI: 0.02–0.28) (Fig. 3).^11^ ^12^ ^13^ ^20^ In terms of midurethral slings, the literature shows a reduction in the incidence of UI and a necessity of surgical treatment for UI postoperatively.^6^ ^11^ ^12^ ^15^ ^16^ ^17^ ^18^ ^19^ ^20^ It was not possible to perform a meta-analysis with the studies by Fuentes^14^ and by Busacchi et al^15^ because their outcomes were different. On the other hand, Burch colposuspension showed more complications and no difference in the incidence of UI postoperatively. (RR = 1.47; 95% CI: 0.28–7.79]) (Fig. 4).^6^ ^16^ ^17^ ^18^

The most common and severe adverse effects are listed on Table 2.

**Table 2:** Complications of prolapse correction surgery and anti-incontinence surgeries

	Author	Intervention	Complications (*n*)				Severe Complications	
			Most frequent complication	*n*	Voiding dysfunction		Type of complications	*n*
					Voiding dysfunction	*De novo* Urgency		
1	Wei et al^11^	POP + TVT	UTI	Intervention: 49/165 Control: 30/172	Voiding dysfunction Intervention: 69/165 Control: 51/172 *p* = 0.02	Intervention: 69/165 Control: 51/172 *p*= 0.02	*	Intervention: 28/165 Control: 20/172
2	Khelaia and Khelaia^12^	AC + TVT	Not informed	−	Not informed	Not informed	Not informed	−
3	Schierlitz et al.^13^	POP + TVT	Urinary retention	Intervention: 2/37 Control: 0/43 p = 0.21	Voiding dysfunction Intervention: 1/37 Control: 1/43 *p* = 0.99	Intervention: 1/37 Control: 3/43 *p* = 0.99	Bleeding (> 500 ml)	Intervention: 2/37 Control: 1/43 *P* = 0.99
4	Fuentes^14^	POP + TVTo	Not informed	−	Not informed	Not informed	Not informed	−
5	Busacchi and Paganotto^15^	AC + TOT	*De novo* urgency	Intervention: 15/50 Control: 17/52	Not informed	Intervention: 15/50 Control: 17/52 24 months = 0.006	Not informed	−
6	Brubaker et al.^16^ ^17^ ^18^	SC + Burch	Irritative symptoms	Intervention:50/157 Control: 58/165 *p* = 0.48	Intervention: 63/157 Control: 66/165 *p* = 0.92	Intervention:22/157 Control: 41/165 *p* = 0.048	**	Intervention: 7/157 Control: 5/165 *P* = 0.79
7	Costantini et al.^6^	SC + Burch	Urinary retention	Intervention: 3/34 Control: 0/32	Intervention: 2/34 Control: 3/32	Intervention: 3/34 Control: 2/32	Blood Transfusion	Intervention: 3/34 Control: 3/32
8	Colombo et al.^19^	CP + PPL	Urinary retention	Intervention:14/50 Control: 12/52 *p =* 0.02	Intervention: 5/50 Control: 0/52 *p* = 0.02	Not informed	Death – bleeding caused by chronic hepatic dysfunction	Intervention: 0/50 Control: 1/52
9	von der Ploeg^20^	POP + MUS	Urinary retention	Intervention: 3/43 Control: 1/48	Intervention: 3/34 Control: 1/32	Intervention: 4/34 Control: 3/32	Urethrolysis for voiding dysfunction	Intervention: 0/43 Control: 1/48

Abbreviations: AC, anterior colporrhaphy; CP, cystopexy; PPL, posterior pubourethral ligaments; POP, pelvic organ prolapse; SC, sacrocolpopexy; SUI, stress urinary incontinence; TOT, out-in transobturator sling; TVT, retropubic sling; TVTo, in-out transobturator sling; UTI, urinary tract infection; y, year.

* A serious adverse event was defined as any untoward medical occurrence (whether or not it was plausibly related to the index surgery) that resulted in death, was life threatening, required inpatient hospitalization, resulted in persistent or serious disability or incapacity, resulted in a congenital anomaly or birth defect, or constituted a medically important condition. An unexpected adverse event was defined as any other untoward event that did not qualify as an expected adverse event. Expected adverse events were defined as common side effects attributable to the placement of a sling. Any expected or unexpected adverse event that qualified as a serious adverse event was counted as such.

** Serious adverse events were defined as untoward medical occurrences that were life-threatening or fatal, required prolonged hospitalization or readmission for the index surgery, any condition that resulted in persistent or clinically significant disability, or any other important medical condition. Since surgical treatment for stress urinary incontinence was a component of the stress-incontinence end point, it was not included among the adverse events.

## Discussion

Nowadays, when it comes to therapeutic interventions, it is recommended that the treatment indication should be based on the best level of scientific evidence, along with the clinical experience of the physician and the expectations of the patient. When focusing specifically on the best level of scientific evidence to determine the effectiveness of an intervention, the systematic review of randomized controlled trials combined with a meta-analysis is the study design that shows the highest level of evidence.^9^ ^21^ Physicians can often find treatment recommendations in narrative reviews and in the discussion sections of articles and of meta-analyses. However, to make a treatment recommendation involves framing a question, identifying and management options, summarizing evidence, and applying value judgments to arrive at an optimal course of action for a certain population. Each step in this process can be conducted systematically, avoiding bias.^22^

In Brazil, due to the gradual aging of the population in the last 50 years, there has been a huge change in the prevalence of diseases. The prevalence of chronic diseases overcame the prevalence of infectious diseases.^7^ In this context, national data estimate that 35% of the female population between 40 and 65 years old will present UI.^7^ ^23^

The Brazilian public healthcare system (SUS, in the Portuguese acronym) is one of the most complex and largest public health systems in the world. It covers from general practice appointments to organ transplantations. The rudiments of the SUS are integral access, universality and gratuitousness to the whole population of the country, which comprises > 180 million people. However, the SUS does not provide national guidelines to treat UI in all cities of the country.^24^

For this reason, a systematic review endorsed by the Urogynecology Committee of the FEBRASGO was conducted to evaluate the impact of anti-incontinence procedures during the surgical correction of prolapse in women with occult SUI. This evaluation included controlled and randomized clinical trials involving a significant number of patients. Our study brings together a more recent systematic review on the treatment of UI in patients with genital prolapse grades 3 and 4. Differently from the meta-analysis performed by Maher et al^25^ and by van der Ploeg et al,^26^ the present study included only randomized clinical trials in which we compared, specifically, trials with genital prolapse correction associated or not with an anti-incontinence procedure. Thus, among the methods studied in our meta-analysis, mid-urethral sling was the only anti-incontinence procedure that reduced postoperative SUI.^11^ ^12^ ^13^ ^14^ ^15^ Regarding transobturator slings, it was not possible to conduct a meta-analysis of the studies by Fuentes^14^ and by Busacchi et al.^15^

In addition, during the correction of stage 3 and 4 prolapses in patients considered asymptomatic from the voiding point of view, it is essential to weigh all the advantages and adverse effects of performing an anti-incontinence procedure. In this sense, Wei et al^11^ showed that the placement of a tension-free medium urethral sling in asymptomatic patients took a rate increased the incidence of intra- and postoperative complications. However, this data is in opposition to numerous studies that did not find additional complications when comparing the correction of prolapse, alone, with combined surgery, employing the mid-urethral sling.^27^ ^28^ Regarding the intraoperative complications of anti-incontinence procedures, the most prevalent event was bladder perforation. However, the evidence in the recent literature informs that more serious complications may arise, such as Retzius space hemorrhage, external iliac vessel injury, intestinal damage, obturator nerve injury, surgical site infections, and extrusion or erosion of meshes.^27^ ^28^ ^29^ ^30^ On the other hand, there are relevant postoperative complications associated with the employment of the sling and of Burch colposuspension, which are urgency *de novo*, and voiding disorders. Urgency *de novo* is characterized as the appearance of UI by urgency after an anti-incontinence surgery. In addition, this diagnosis is only valid for patients who do not present symptoms of urge-incontinence in the preoperative period. Therefore, the purpose of the present review was to inform adequately the symptoms of postoperative micturition disorders (Table 2), considering that these events should be counted as complications of the procedure. It is known that prevention of SUI is an important goal in the surgical treatment of pelvic organ prolapse. However, the emergence of another problem secondary to this treatment can lead to postoperative discomfort, as well as decrease long-term patient satisfaction.^29^ ^30^ Finally, another point to be considered was the fact that the procedures for correction of genital prolapse varied widely between studies, which may have influenced the effectiveness of the treatment. In addition, these studies did not have a follow-up to evaluate the efficacy of the method. These aforementioned points could help the design of a protocol for future studies.

Another important issue to discuss is that with the advances in artificial intelligence applied in medicine, computers will soon perform the systematic review and humans will only have to analyze the data to apply on the patients.

## Conclusion

We have concluded that performing any prophylactic anti-incontinence procedure at the same time of prolapse repair reduced the incidence of SUI postoperatively. The midurethral sling procedure reduced the incidence of surgical treatment for SUI postoperatively. The Burch colposuspension did not show any decrease in the incidence of SUI postoperatively.
